# Relationship between higher-order wavefront aberrations and natural progression of myopia in schoolchildren

**DOI:** 10.1038/s41598-017-08177-6

**Published:** 2017-08-11

**Authors:** Takahiro Hiraoka, Junko Kotsuka, Tetsuhiko Kakita, Fumiki Okamoto, Tetsuro Oshika

**Affiliations:** 1 Department of Ophthalmology, Faculty of Medicine, University of Tsukuba, Ibaraki, Japan; 2 Kakita Eye Clinic, Chiba, Japan

## Abstract

This study investigated the relationship between higher-order aberrations (HOAs) and myopia progression as well as axial elongation in schoolchildren. We examined cycloplegic refraction, axial length, and wavefront aberrations prospectively in 71 myopic children. Changes in cycloplegic refraction and axial length during a 2-year study period were assessed, and their correlations with HOA components were analyzed. Sixty-four subjects ([mean ± SD] 9.2 ± 1.6 years) completed the 2-year examinations. Cycloplegic refraction was significantly changed after 2 years (P < 0.0001), and the average change (myopia progression) was −1.60 ± 1.04 D. Axial length also increased significantly (P < 0.0001), and the average increase (axial elongation) was 0.77 ± 0.40 mm. Myopia progression and axial elongation showed significant correlations with many components of corneal HOA (P < 0.0001 to P = 0.0270). Multivariate analysis showed that the total HOA of the cornea was the most relevant variable to myopia progression and axial elongation (P < 0.0001). Eyes with larger amounts of corneal HOAs showed less myopia progression and smaller axial elongation, suggesting that corneal HOAs play a role in the refractive and ocular developments in children.

## Introduction

Myopia is one of the leading causes of vision impairment worldwide. The prevalence of myopia has risen steeply over the past 50 years especially in developed countries in east and southeast Asia, such as China, Taiwan, Hong Kong, South Korea, Japan, and Singapore^1–3^. An increased prevalence of myopia has also been confirmed in the United States^4^. Myopia progression and axial elongation are irreversible and associated with increased risks of ocular complications such as chorioretinal degeneration, retinal detachment, glaucoma, and cataract^5–9^. The recent dramatic increases in myopia rates have made it a major public health problem^4, 10^ and a considerable socioeconomic burden^11, 12^. Myopia is thought to have a multifactorial etiology^1–3^, including both genetic and environmental factors. However, understanding of the factors associated with its progression is limited.

Thus far, there have been several prevention strategies for myopia progression and axial elongation. The optical approach is one such strategy, and many researchers have tried to prevent myopia progression by altering the optical properties of the eye with progressive addition spectacles^13–15^, multifocal soft contact lenses (SCLs)^16–19^, and orthokeratology^20–23^. Many studies over the last decade have confirmed the effect of overnight orthokeratology in slowing axial elongation and thus preventing myopia progression^20–23^. Additionally, meta-analyses and systematic reviews of previous studies have shown the considerable efficacy of orthokeratology in controlling axial elongation in myopic children^24–27^, although the mechanism remains unknown.

Recently, we examined the influence of higher-order wavefront aberrations (HOAs) on axial elongation in myopic children treated with orthokeratology, and found a significant negative correlation between coma-like aberration and axial elongation^28^. This implies that asymmetric HOAs such as coma inhibit axial elongation. Additionally, it has been reported that SCLs with positive spherical aberration might slow myopia progression^29^. Based on these findings, we believed that the effect of HOAs on myopia progression and axial elongation should be investigated over the natural course of childhood myopia. However, little is known about this effect. We therefore conducted the current study to investigate the relationship between HOAs, myopia progression, and axial elongation in normal schoolchildren with mild to moderate myopia.

## Results

Of the 71 children initially enrolled in the study, 7 dropped out because of relocation to another city, strong desire to use contact lenses, or lack of motivation to keep follow-up appointments. We successfully completed the 2-year follow-up examinations in 64 children. Their demographics are summarized in Table 1. There were 36 boys and 28 girls, and their ages ranged from 6 to 12 (9.2 ± 1.6, mean ± standard deviation). Cycloplegic refraction (spherical equivalent refractive error) ranged from −4.30 to −1.51 dioptre (D) (−2.73 ± 0.74 D). LogMAR uncorrected visual acuity ranged from 0.40 to 1.40 (0.86 ± 0.19), and logMAR best-corrected visual acuity ranged from −0.18 to –0.08 (−0.13 ± 0.05). Axial length ranged from 22.72 to 25.88 mm (24.58 ± 0.73 mm).

**Table 1:** Baseline demographic information and the corneal and ocular HOAs in those subjects who completed the 2-year examinations.

	Mean ± Standard deviation	Range	
		Min	Max
Age (years)	9.2 ± 1.6	6	12
SER (D)	−2.73 ± 0.74	−4.30	−1.51
UCVA (logMAR)	0.86 ± 0.19	0.40	1.40
BCVA (logMAR)	−0.13 ± 0.05	−0.18	−0.08
Axial length (mm)	24.58 ± 0.73	22.72	25.88
Corneal HOA (μm)			
*C*_3_^−1^	−0.096 ± 0.174	−0.517	0.516
*C*_3_^1^	−0.153 ± 0.101	−0.398	0.105
*C*_4_°	0.221 ± 0.081	0.051	0.461
*C*_3_^−3^	−0.036 ± 0.076	−0.249	0.090
*C*_3_^3^	−0.022 ± 0.084	−0.199	0.136
*C*_4_^−4^	0.013 ± 0.033	−0.062	0.087
*C*_4_^−2^	−0.014 ± 0.023	−0.068	0.041
*C*_4_^2^	−0.018 ± 0.062	−0.173	0.131
*C*_4_^4^	−0.011 ± 0.037	−0.104	0.075
S3	0.289 ± 0.113	0.110	0.577
S4	0.246 ± 0.084	0.096	0.533
S5	0.059 ± 0.029	0.018	0.218
S6	0.045 ± 0.028	0.017	0.197
S3 + 5	0.296 ± 0.113	0.123	0.585
S4 + 6	0.251 ± 0.086	0.100	0.571
S3 + 4 + 5 + 6	0.399 ± 0.112	0.219	0.815
Ocular HOA (μm)			
*C*_3_^−1^	0.111 ± 0.185	−0.289	0.677
*C*_3_^1^	0.014 ± 0.109	−0.195	0.248
*C*_4_^0^	0.068 ± 0.118	−0.165	0.331
*C*_3_^−3^	−0.046 ± 0.119	−0.303	0.218
*C*_3_^3^	0.001 ± 0.118	−0.298	0.194
*C*_4_^−4^	0.023 ± 0.036	−0.060	0.098
*C*_4_^−2^	−0.022 ± 0.026	−0.097	0.037
*C*_4_^2^	0.008 ± 0.064	−0.151	0.167
*C*_4_^4^	0.001 ± 0.004	−0.102	0.007
S3	0.280 ± 0.135	0.086	0.742
S4	0.162 ± 0.071	0.047	0.341
S5	0.062 ± 0.030	0.025	0.183
S6	0.051 ± 0.017	0.016	0.102
S3 + 5	0.289 ± 0.136	0.098	0.759
S4 + 6	0.173 ± 0.068	0.065	0.343
S3 + 4 + 5 + 6	0.345 ± 0.133	0.154	0.807

SER = spherical equivalent refraction; UCVA = uncorrected visual acuity; BCVA = best-corrected visual acuity; logMAR = logarithm of the minimum angle of resolution; HOA = higher-order aberration; *C*_3_^−1^ *= *vertical coma aberration; *C*_3_^1^ *= *horizontal coma aberration; *C*_4_^0^ = spherical aberration; *C*_3_^−3^, *C*_3_^3^ = trefoils; *C*_4_^−4^, *C*_4_^4^ = tetrafoils; *C*_4_^−2^, *C*_4_^2^ = secondary astigmatisms; S3 = 3rd-order RMS aberrations; S4 = 4th-order RMS aberrations; S5 = 5th-order RMS aberrations; S6 = 6th-order RMS aberrations; S3 + 5 = coma-like aberrations; S4 + 6 = spherical-like aberrations; S3 + 4 + 5 + 6 = total higher-order aberrations.

Cycloplegic refraction changed significantly from −2.73 ± 0.74 D to −4.33 ± 1.21 D over the 2-year study (P < 0.0001, paired *t*-test). The change (myopia progression) was –1.60 ± 1.04 D. Over the same period, axial length increased significantly from 24.58 ± 0.73 to 25.35 ± 0.82 mm (P < 0.0001). The increase (axial elongation) was 0.77 ± 0.40 mm.

Myopia progression and axial elongation were analyzed in relation to the HOA components. Myopia progression showed significant simple correlations with all the components of corneal HOA (Pearson correlation coefficient; *r* = –0.292 to 0.546, P < 0.0001 to = 0.0361) except *C*_3_^−3^, *C*_3_^3^, *C*_4_^−2^, and *C*_4_^4^ (*r* = –0.079 to 0.098, P = 0.4446 to 0.8442) (Table 2). Axial elongation also exhibited significant simple correlations with all the components of corneal HOA (*r* = −0.584 to 0.305, P < 0.0001 to = 0.0270) except *C*_3_^−3^, *C*_3_^3^, *C*_4_^−4^, *C*_4_^−2^, and *C*_4_^4^ (*r* = −0.222 to 0.147, P = 0.0774 to 0.5099) (Table 3). Myopia progression and axial elongation also showed significant correlations with many components of ocular HOA (*r* = −0.371 to 0.365, P = 0.0023 to 0.1691), but the correlations were apparently weaker than those with corneal HOA components (Tables 2 and 3). Additionally, myopia progression and axial elongation were significantly correlated with initial age (*r* = 0.356, P = 0.0036; and *r* = −0.489, P < 0.0001, respectively), but not with initial spherical equivalent refractive error (*r* = −0.112, P = 0.3781; and *r* = 0.109, P = 0.3938, respectively), astigmatism (*r* = −0.078, P = 0.5430; and *r* = 0.088, P = 0.4914, respectively), or initial axial length (*r* = −0.006, P = 0.9644; and *r* = −0.029, P = 0.8187, respectively).

**Table 2:** Results of univariate analysis between averaged HOA components and myopia progression.

Corneal HOA	Correlation coefficient	P-value	Ocular HOA	Correlation coefficient	P-value
*C*_3_^−1^	0.293	0.0186*	*C*_3_^−1^	0.362	0.0030*
*C*_3_^1^	−0.292	0.0187*	*C*_3_^1^	−0.294	0.0178*
*C*_4_^0^	0.349	0.0045*	*C*_4_^0^	0.228	0.0705
*C*_3_^−3^	−0.076	0.5499	*C*_3_^−3^	−0.044	0.7288
*C*_3_^3^	−0.079	0.5364	*C*_3_^3^	−0.084	0.5132
*C*_4_^−4^	0.262	0.0361*	*C*_4_^−4^	−0.005	0.9705
*C*_4_^−2^	0.025	0.8442	*C*_4_^−2^	0.141	0.2677
*C*_4_^2^	−0.288	0.0208*	*C*_4_^2^	−0.134	0.2924
*C*_4_^4^	0.098	0.4446	*C*_4_^4^	−0.046	0.7207
S3	0.407	0.0007*	S3	0.342	0.0053*
S4	0.432	0.0003*	S4	0.199	0.1145
S5	0.398	0.0010*	S5	0.334	0.0067*
S6	0.418	0.0005*	S6	0.164	0.1966
S3 + 5	0.415	0.0006*	S3 + 5	0.350	0.0043*
S4 + 6	0.438	0.0002*	S4 + 6	0.204	0.1056
S3 + 4 + 5 + 6	0.546	<0.0001*	S3 + 4 + 5 + 6	0.365	0.0028*

HOA = higher-order aberration; *C*_3_^−1^ = vertical coma aberration; *C*_3_^1^ *= *horizontal coma aberration; *C*_4_^0^ = spherical aberration; *C*_3_^−3^, *C*_3_^3^ = trefoils; *C*_4_^−4^, *C*_4_^4^ = tetrafoils; *C*_4_^−2^, *C*_4_^2^ = secondary astigmatisms; S3 = 3rd-order RMS aberrations; S4 = 4th-order RMS aberrations; S5 = 5th-order RMS aberrations; S6 = 6th-order RMS aberrations; S3 + 5 = coma-like aberrations; S4 + 6 = spherical-like aberrations; S3 + 4 + 5 + 6 = total higher-order aberrations. *Significant correlation by the Pearson correlation test.

**Table 3:** Results of univariate analysis between averaged HOA components and axial elongation.

Corneal HOA	Correlation coefficient	P-value	Ocular HOA	Correlation coefficient	P-value
*C*_3_^−1^	−0.276	0.0270*	*C*_3_^−1^	−0.357	0.0035*
*C*_3_^1^	0.305	0.0140*	*C*_3_^1^	0.295	0.0175*
*C*_4_^0^	−0.431	0.0003*	*C*_4_^0^	−0.332	0.0071*
*C*_3_^−3^	0.102	0.4240	*C*_3_^−3^	0.108	0.3970
*C*_3_^3^	0.147	0.2469	*C*_3_^3^	0.127	0.3184
*C*_4_^−4^	−0.222	0.0774	*C*_4_^−4^	−0.026	0.8361
*C*_4_^−2^	−0.084	0.5099	*C*_4_^−2^	−0.087	0.4980
*C*_4_^2^	0.347	0.0047*	*C*_4_^2^	0.238	0.0580
*C*_4_^4^	−0.133	0.2964	*C*_4_^4^	−0.002	0.9894
S3	−0.384	0.0016*	S3	−0.308	0.0128*
S4	−0.511	<0.0001*	S4	−0.302	0.0148*
S5	−0.427	0.0004*	S5	−0.347	0.0046*
S6	−0.428	0.0004*	S6	−0.174	0.1691
S3 + 5	−0.395	0.0011*	S3 + 5	−0.317	0.0104*
S4 + 6	−0.516	<0.0001*	S4 + 6	−0.307	0.0132*
S3 + 4 + 5 + 6	−0.584	<0.0001*	S3 + 4 + 5 + 6	−0.371	0.0023*

HOA = higher-order aberration; *C*_3_^−1^ = vertical coma aberration; *C*_3_^1^ *= *horizontal coma aberration; *C*_4_^0^ = spherical aberration; *C*_3_^−3^, *C*_3_^3^ = trefoils; *C*_4_^−4^, *C*_4_^4^ = tetrafoils; *C*_4_^−2^, *C*_4_^2^ = secondary astigmatisms; S3 = 3rd-order RMS aberrations; S4 = 4th-order RMS aberrations; S5 = 5th-order RMS aberrations; S6 = 6th-order RMS aberrations; S3 + 5 = coma-like aberrations; S4 + 6 = spherical-like aberrations; S3 + 4 + 5 + 6 = total higher-order aberrations. *Significant correlation by the Pearson correlation test.

**Figure 1:**
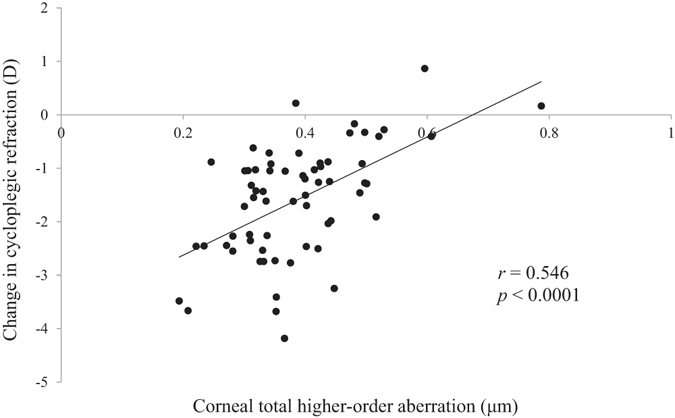
Scatterplots showing the averaged corneal total HOAs vs the change in cycloplegic refraction over 2 years. There was a significant correlation between the parameters (Pearson correlation coefficient; *r* = 0.546, P < 0.0001), D = dioptre.

**Figure 2:**
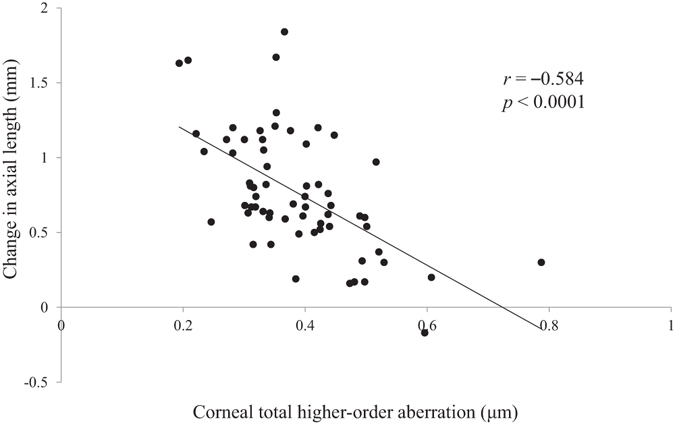
Scatterplots showing the averaged corneal total HOAs vs the change in axial length over 2 years. There was a significant correlation between the parameters (Pearson correlation coefficient; *r* = −0.584, P < 0.0001).

**Figure 3:**
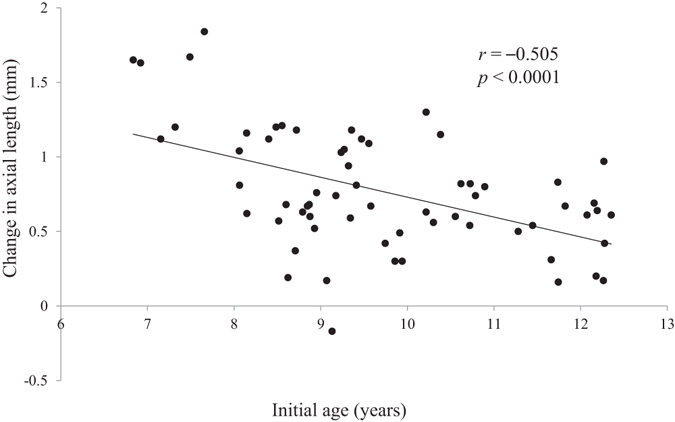
Scatterplots showing the initial age vs the change in axial length over 2 years. There was a significant correlation between the parameters (Pearson correlation coefficient; *r* = −0.505, P < 0.0001).

Thereafter, we employed multivariate analysis with stepwise regression to identify the factors that directly influenced myopia progression and axial elongation, because several parameters can interrelate with each other, leading to spurious correlations. The total HOA of the cornea (3rd + 4th + 5th + 6th-order aberration) was identified as the only independent predictive factor for myopia progression (P < 0.0001; standard regression coefficient = 0.546) (Table 4, Fig. 1). As for axial elongation, the total HOA of the cornea and age were independently correlated with axial elongation (both P < 0.0001; standard regression coefficient = –0.453 and –0.320, respectively) (Table 4 and Figs 2 and 3), with the total HOA of the cornea being the more relevant variable. Similarly, when the initial visit data were used for multivariate analysis instead of the average data of all visits, almost the same results were confirmed, as follows; the total HOA of the cornea and ocular *C*_3_^−1^ were identified as independent predictive factors for myopia progression (both P < 0.0001; standard regression coefficient = 0.435 and 0.260, respectively), with the total HOA of the cornea being the more relevant variable. As for axial elongation, the total HOA of the cornea and age were independently correlated with axial elongation (both P < 0.0001; standard regression coefficient = −0.390 and −0.329, respectively), with the total HOA of the cornea being the more relevant variable.

**Table 4:** Factors affecting myopia progression and axial elongation in multiple regression analysis.

Response variable	Explanatory variable	P-value	Standard regression coefficient	Overall *R* ^2^
Myopia progression	Total HOA of the cornea	<0.0001	0.546	0.298
Axial elongation	Total HOA of the cornea age	<0.0001	−0.453	0.427
		<0.0001	−0.320	

HOA = higher-order aberration.

## Discussion

This 2-year prospective study showed that HOAs are strongly correlated with myopia progression and axial elongation in myopic schoolchildren wearing single-vision spectacles. This is the first study to elucidate such relationships in the natural course of refractive development. Additionally, the multivariate analysis showed that the total HOA of the cornea is more relevant to myopia progression and axial elongation than other parameters including age. It is well known that age is an important factor in myopia progression^30–32^. Hyman *et al*.^32^. concluded that a younger initial age was the strongest factor independently related to myopic progression and axial elongation in their 3-year prospective study. This association was confirmed in our current and previous studies^28^, but both studies showed that the effect of HOAs on ocular growth and refractive development was greater than that of age.

In our study, the mean root mean square (RMS) of ocular total HOAs for a 6-mm pupil was 0.355 ± 0.133 μm. Several studies have examined HOAs in children of age ranges similar to our study group. Kirwan *et al*.^33^. reported that the mean RMS of ocular total HOAs for a 6-mm pupil was 0.462 ± 0.100 μm in myopic children with a mean age of 6.7 years. In our previous study^34^, it was 0.304 ± 0.096 μm in myopic children with a mean age of 7.3 years. The current results are between those. Considering other refractive errors, Kirwan *et al*.^33^. showed that it was 0.357 ± 0.131 μm in hyperopic children, which is quite similar to the current results from myopic children. Numerous studies have investigated the relationship between monochromatic aberrations and refractive status. Some studies^33, 35, 36^ suggested that myopic eyes had greater levels of HOAs, but other studies^37–39^ found no differences in aberration characteristics in myopic eyes, emmetropic eyes, and hyperopic eyes. Although this variation might be attributed to differences in sample size, ethnicity, and age, no definitive conclusions have been drawn yet. Additionally, there is much intersubject variability in HOAs^34, 38^. Indeed, much intersubject variability in the magnitude of HOAs was observed in this study.

In recent years, it has been shown that various types of multifocal SCLs can retard the rate of myopia progression and axial elongation^16–19^. Notably, similar efficacy was confirmed in each study even though the employed SCLs had very different designs. Sankaridurg *et al*.^17^. showed the effect of a novel SCL designed to reduce relative peripheral hyperopia on the basis of the “peripheral refraction theory,” which states that peripheral hyperopic retinal blur is an important trigger for axial elongation and myopia progression^40–42^. The SCL had a central zone for correcting refractive errors and a peripheral zone with progressively increasing positive power of up to +2.0 D, and the study showed that myopia progression and axial elongation over a 12-month period were 34% and 33% lower, respectively, in the SCL group than in the control group wearing conventional single-vision spectacles. However, Ticak and Walline^43^ examined the peripheral optical profiles of a similar multifocal SCL with a distance-centre design and a +2.00 D positive power in the periphery and showed that there was almost no peripheral myopic shift. Thus, the control mechanism of myopia progression cannot be fully explained by the “peripheral refraction theory” alone.

Another proposal is the “accommodation lag theory,” which assumes that the axial hyperopic retinal blur due to high accommodation lag during near-vision work accelerates eye growth^44, 45^. Based on this theory, several studies have been conducted using centre-distance bifocal SCLs designed to correct accommodation lag^16, 18, 19^. Anstice and Phillips^18^ tested bifocal SCLs featuring a central correction zone surrounded by a series of treatment (+2.00 D addition) and correction zones that together produced 2 focal planes. They reported that these SCLs reduced myopia progression and axial elongation during the first year by 37% and 49%, respectively, compared to single-vision lenses. More recently, Fujikado *et al*.^19^. reported that a low-addition (+0.50 D peripherally) SCL with a decentred optical design reduced axial elongation by 47% after 12 months in myopic children. It should be noted that a SCL with a different additional power and design also showed an inhibitory effect on axial elongation similar to the above-mentioned multifocal SCLs.

It is necessary to consider how HOAs act in slowing axial elongation and myopia progression. In pseudophakic eyes, a significant positive correlation was observed between coma-like aberration and apparent accommodation^46^. Similarly, in eyes undergoing excimer laser corneal surgery, corneal HOAs were reported to be associated with corneal pseudoaccommodation^47^. It is also known that HOAs can increase the depth-of-focus of the eye^47, 48^. It is therefore likely that retinal blur caused by accommodation lag during near-vision work is improved by the presence of large corneal HOAs, thereby inhibiting excessive eye growth consistent with the “accommodation lag theory”. It is also known that increased depth-of-focus allows the accommodative mechanism to exert the minimum necessary accommodation amplitude to bring the stimulus into focus^49^. Large HOAs might therefore increase the apparent accommodation and/or depth-of-focus, thereby reducing the accommodative effort of ciliary muscles, especially in near-vision tasks. The “mechanical tension hypothesis” is one proposed explanation for axial elongation and myopia progression; it hypothesizes that the mechanical tension created by the ciliary body and crystalline lens during accommodation, which causes forward and inward choroid pulling, restricts ocular growth within the eye’s equatorial dimension, ultimately causing accelerated axial elongation^50, 51^. Indeed, it has been shown that both animal^52^ and human eyes^50, 53^ elongate during accommodation. Hence, in eyes that perform more near-vision work, the ciliary muscle will contract more frequently and for a greater length of time, which may predispose the eye to long-term growth changes^54^. Based on this hypothesis, a reduced accommodative effort owing to larger HOAs lessens mechanical tension at the equator (within the equatorial dimension) and allows more proportional eye expansion, ultimately leading to slower axial elongation. In fact, in our previous study, various HOA components showed significant negative correlations with axial elongation in myopic eyes undergoing orthokeratology^28^.

However, there have been several studies that negate the relationship between accommodation and myopia progression^55, 56^. Unfortunately, one limitation of our study was that accommodative responses were not evaluated. Further study should be conducted to clarify the relationship between accommodation, HOAs, and myopia progression. Additionally, only myopic children were enrolled in this study. Another study including emmetropic children should be conducted to determine whether HOAs have a more general role in the etiology of myopia.

In conclusion, we found that both corneal and ocular HOAs were negatively correlated with myopia progression and axial elongation in myopic schoolchildren and that the associations with corneal HOAs were stronger than those with ocular HOAs. Although several HOA components were significantly correlated with myopia progression and axial elongation, total corneal HOA (combined aberrations from third- to sixth-order) was the most relevant factor. Including aberrometry in vision screenings for preschool- and school-aged children may help predict future myopia progression. Additionally, modification or control of corneal HOAs with specially-designed SCLs may become a promising strategy for the prevention of myopia progression in children. The current study demonstrates the need for further investigation to clarify the role of HOAs in refractive development.

## Methods

### Subjects

This was a prospective, noncomparative study designed to evaluate the effect of HOAs on the progression of juvenile-onset myopia. Myopic children were invited to participate in this study if they satisfied our eligibility criteria (Table 5). All study protocols conformed to the Declaration of Helsinki and the institutional review board of University of Tsukuba Hospital approved the research protocols. Written informed consent was obtained from all parents and written assent from all children after a written and verbal explanation of the clinical procedures.

**Table 5:** Eligibility Criteria.

1. Ages from 6 to 12 years at the start of the study
2. Cycloplegic autorefraction (spherical equivalent) from −4.50 to −1.50 D in both eyes
3. Astigmatism (cycloplegic autorefraction) ≤ 1.50 D in both eyes
4. Anisometropia (cycloplegic autorefraction) ≤ 1.50 D
5. Best-corrected visual acuity ≥ 0.00 logMAR units in both eyes (equivalent to Snellen 20/20)
6. No strabismus or other ocular diseases except refractive error
7. No systemic diseases that might affect refractive development
8. No use of medications that might affect refractive development
9. No history of wearing bifocal or progressive addition spectacles
10. No history of orthokeratology or use of contact lenses

D = dioptre, logMAR = logarithm of the minimum angle of resolution.

Seventy-one subjects who fulfilled the eligibility criteria were enrolled in this study. They were required to return for follow-up examinations every 6 months during the 2-year study period. Visual acuity, cycloplegic refraction, axial length, and wavefront aberrations were evaluated at each visit. For all subjects, new single-vision spectacles were prescribed by a certified ophthalmic technician at the time of enrolment and were replaced throughout the study period if visual acuity with the spectacle correction was found to be less than 20/20.

### Refraction Measurements

Cycloplegia was induced with three drops of 1% cyclopentolate hydrochloride (Cyplegin^®^ 1% ophthalmic solution; Santen Pharmaceutical Co., Ltd., Osaka, Japan) at 5-min intervals, and auto-refractometry was done 60 min after the first instillation using an auto ref-keratometer (RT-7000; Tomey Co., Nagoya, Japan) with a 0.01-D scale. Adequate pupil dilation and unresponsiveness to light were confirmed before measurements. Five measurements were made for each eye and averaged for the following analyses.

### Axial Length Measurements

Axial length was evaluated using noncontact measurements with a partial coherence interferometer (IOLMaster; Carl Zeiss Meditec, Dublin, CA, USA) that can provide successive, repeatable, high-resolution measurements^57^. At each visit, 10 successive measurements were taken, and their average was used as a representative value.

### Higher-Order Aberration Measurements

Corneal and ocular higher-order aberrations for a 6-mm pupil were simultaneously measured with a Hartmann–Shack wavefront analyzer with Placido disk topographer (KR-1W; Topcon Co., Tokyo, Japan) after cycloplegia^58^. The acquired data sets were expanded with the normalized Zernike polynomials, and root mean square (RMS; in μm) values from third- to sixth-order Zernike coefficients were calculated based on a common pupil size of 6.0 mm. Aberrations higher than 3rd-order cannot be corrected by spectacles, and are thus identified as higher-order aberrations. The polynomials can be expanded up to any arbitrary order if a sufficient number of measurements are made for the calculations, but usually Zernike coefficients up to 4th- or 6th-order are used in clinical practice. From these Zernike coefficients, 3rd-order aberration (S3), 4th-order aberration (S4), 5th-order aberration (S5), 6th -order aberration (S6), coma-like aberrations (S3 + S5), spherical-like aberrations (S4 + S6), and total HOAs (S3 + S4 + S5 + S6) were calculated. Coma-like and spherical-like aberrations are representative components of HOA, and consist of asymmetric and symmetric Zernike coefficients, respectively. Total HOAs (the square root of the sum of squares of terms from third- to sixth-order) can be used to estimate the severity of optical quality deterioration for diagnostic purposes. S3 has 4 individual terms that represent coma (*C*_3_^−1^ and *C*_3_^1^) and trefoil (*C*_3_^−3^ and *C*_3_^3^), and S4 similarly has 5 individual terms that represent tetrafoil (*C*_4_^−4^ and *C*_4_^4^), secondary astigmatism (*C*_4_^−2^ and *C*_4_^2^) and spherical aberration (*C*_4_^0^). S5 has 6 individual terms (*C*_5_^−5^ to *C*_5_^5^), and S6 has 7 individual terms (*C*_6_^−6^ to *C*_6_^6^). Among these individual terms, coma (*C*_3_^−1^ and *C*_3_^1^) and spherical (*C*_4_^0^) aberrations are proven to considerably affect vision quality^59^. The measurements were repeated at least 5 times for each eye, 3 well-focused images were selected, and the coefficients determined from each image were averaged. The averaged values were used for the subsequent analyses.

### Statistical Analysis

Only data from the right eyes were used for analyses, because optical parameters are very similar between the left and right eyes of the same subject^60^. Data obtained at the 2-year visit were compared with the initial measurements using the paired *t*-test. For each HOA component, data from 5 visits during the study period (0, 6, 12, 18, and 24 months) were averaged and then used for univariate analyses between the HOA components and myopia progression, as well as between HOA components and axial elongation using the Pearson correlation test. In this study, myopia progression and axial elongation were defined as changes in cycloplegic autorefraction and axial length, respectively, during the 2-year study period. Thereafter, multivariate analysis with stepwise regression was performed using the forward selection technique to identify explanatory variables with a statistically significant contribution to myopia progression and axial elongation. All statistical analyses were performed using StatView (version 5.0; SAS Institute Inc., Cary, NC, USA). P < 0.05 was considered significant.
